# Precision-Engineered CsPbBr_3_ Quantum Dots with Encapsulation and Tunable Size Exhibiting Temperature-Responsive Luminescence for Emerging Sensors

**DOI:** 10.3390/mi17101174

**Published:** 2026-10-10

**Authors:** Amir Sohail, Aleksandr Tarasov, Aleksandr Degterev, Mariya Degtereva, Marina Pavlova, Nikita Khorshev, Yevgeniy Levin, Ivan Lamkin, Sergey Tarasov

**Affiliations:** Photonics Department, Saint Petersburg Electrotechnical University “LETI”, 197022 Saint Petersburg, Russia

**Keywords:** perovskite quantum dots, CsPbBr_3_, hot injection, photoluminescence, polymer encapsulation

## Abstract

In this work, CsPbBr_3_ perovskite quantum dots (QDs) were synthesised by hot injection, with reaction time controlling nanocrystal size and emission from 493 to 515 nm. Temperature-dependent photoluminescence (23–80 °C) showed decreasing intensity but stable peak position. Embedding QDs in PMMA and PVK revealed polymer-dependent thermal behaviour: insulating PMMA preserved radiative recombination and gave stable, reproducible quenching, while semiconducting PVK introduced interfacial charge transfer, enhancing thermal sensitivity. The results establish CsPbBr_3_ QD–polymer composites as tunable temperature-sensitive platforms for optical sensing.

## 1. Introduction

In the last decade, all-inorganic caesium lead bromide perovskite quantum dots (CsPbBr_3_ QDs) have emerged as one of the most intensively studied classes of semiconductor nanocrystals due to their excellent optical and electronic properties. They exhibit near-unity photoluminescence quantum yields (PLQYs), spectrally narrow emission linewidths <20 nm, high colour purity and large bandgap tunability across the visible spectrum by precise control of nanocrystal size and halide composition [1]. These properties make CsPbBr_3_ QDs very attractive for a wide range of optoelectronic applications such as light-emitting diodes (LEDs), solid-state lasers, photodetectors, luminescent solar concentrators and advanced optical sensing platforms [1,2]. All-inorganic CsPbBr_3_ QDs, in comparison to conventional II–VI semiconductor quantum dots such as cadmium selenide (CdSe) or III–V systems such as indium phosphide (InP), have the additional advantages of facile solution-processable synthesis, higher defect tolerance due to the shallow nature of their intrinsic defect states, and a soft ionic lattice that can tolerate a compositional substitution with little disruption to the structure [2,3].

The quantum confinement effects, which happen when the nanocrystal size is smaller than the exciton Bohr radius (∼7 nm for bulk CsPbBr_3_) [4], are inevitably important to the optical properties of CsPbBr_3_ QDs. This regime leads to a discrete energy level structure and a large blue-shift of the bandgap with respect to the bulk value due to the spatial confinement of charge carriers, enabling continuous and predictable tuning of the emission wavelength by controlling nanocrystal size. The hot-injection method is still the most commonly used synthetic strategy to produce CsPbBr_3_ QDs with high crystallinity, controlled size and narrow size distribution [5,6]. In this method, a fast injection of a precursor of caesium oleate into a hot solution of lead bromide in the presence of coordinating ligands initiates a burst nucleation event, followed by nanocrystal growth under thermodynamic control. Nucleation and growth kinetics can be effectively controlled by systematically varying reaction time, temperature, and precursor stoichiometry, which provides reproducible tuning of nanocrystal size, emission wavelength, and photoluminescence (PL) intensity [7].

Although CsPbBr_3_ QDs exhibit excellent optoelectronic performance, the intrinsic instability under ambient conditions remains a significant barrier to their practical applications. These nanocrystals are highly susceptible to structural degradation upon exposure to moisture, molecular oxygen, polar solvents, high temperatures, and prolonged UV illumination because of the ionic nature of the perovskite crystal lattice [6]. The presence of surface trap states due to halide vacancies, undercoordinated lead ions and incomplete passivation of the nanocrystal surface by oleate and oleylamine ligands is one of the main causes of optical degradation [5]. These surface defects act as non-radiative recombination centres which decrease the PLQY and increase the photodegradation rate. Additionally, the dynamic and labile nature of the coordinative ligand shell results in progressive ligand desorption under ambient or elevated temperature conditions, exposing reactive surface sites and further degrading both optical performance and environmental durability [5,6]. Together, these instability mechanisms represent a major bottleneck for the transition of perovskite QD technology from laboratory demonstration to commercial deployment.

Of these, polymer encapsulation has emerged as one of the most effective, scalable and technologically compatible approaches to improve the stability of perovskite QDs without affecting their intrinsic photophysical properties [8]. The QDs are embedded in a solid polymer matrix that isolates the nanocrystals from environmental conditions, limits ligand desorption by steric confinement, and partially passivates surface trap states through host–guest interactions. Poly(methyl methacrylate) (PMMA) is one of the most widely studied insulating polymer hosts for this purpose. PMMA exhibits excellent optical transparency in the visible and near-ultraviolet spectral regions, a high glass transition temperature (~105 °C), strong moisture barrier properties, and is electrically insulating, which quenches charge-mediated non-radiative pathways [9,10]. Poly (N-vinylcarbazole) (PVK), on the other hand, is a well-known hole-transporting semiconducting polymer with a large bandgap and high thermal stability and has been widely used as a host matrix in organic LEDs and hybrid electroluminescent devices [11]. The incorporation of CsPbBr_3_ QDs in PVK enables charge transfer interactions at the polymer–nanocrystal interface, which help carrier injection in electroluminescent architectures but also provide additional non-radiative dissipation channels. The fundamental difference between these two polymer environments, one being a completely insulating medium and the other electronically active, provides a useful and experimentally controlled system to probe the role of polymer–QD interfacial interactions in thermal stability and luminescence behaviour.

Temperature-dependent PL spectroscopy is a powerful and widely used diagnostic technique to probe the carrier recombination dynamics, exciton–phonon coupling strength and thermal stability of quantum-confined semiconductor systems [4]. In CsPbBr_3_ QDs, the increased temperature activates phonon-assisted non-radiative recombination processes, leading to the progressive quenching of the PL intensity, spectral broadening and, in some material systems, a temperature-induced red-shift of the emission peak associated with the lattice thermal expansion and bandgap renormalisation [12,13]. Non-contact luminescent thermometry, which is gaining importance for thermal mapping in biological microenvironments, integrated photonic circuits, and flexible electronic systems where conventional contact-based methods are impractical [14,15], is based on the temperature sensitivity of luminescence intensity. Crucially, the thermal PL response of polymer-embedded QD composites should be markedly different from that of colloidal nanocrystals, because the polymer matrix changes the local dielectric environment, restricts the nanocrystal surface dynamics, and can either introduce or suppress non-radiative pathways based on the electronic character and thermal conductivity of the polymer [16,17]. While important advances have been achieved individually in size-controlled QD synthesis, polymer matrix engineering, and temperature-resolved spectroscopy, the combination of all three in one comparative experimental study is rarely reported.

In this article, CsPbBr_3_ QDs are synthesised by the hot-injection method, and nanocrystal sizes are systematically controlled by tuning the reaction time and the precursor stoichiometry, enabling direct bandgap engineering in the blue–green spectral region. This is followed by room-temperature optical characterisation and temperature-dependent PL measurements in the 23–80 °C temperature range to study the thermal activation of the non-radiative recombination and its effect on the emission intensity and spectral stability. Next, QDs are embedded into PMMA and PVK polymer matrices, and the thermal PL behaviour of each composite system is systematically studied and compared to that of bare QDs. This study offers a mechanistic understanding of how the polymer host influences the thermal optical stability and non-radiative carrier dynamics by correlating the polymer’s electronic structure with the observed luminescence response. The results reveal that CsPbBr_3_ QD–polymer composites constitute a promising and scalable platform for non-contact luminescent thermometry, wavelength-selective photonic components and thermally stable flexible optoelectronic devices [18,19].

## 2. Materials and Methods

### 2.1. Materials

Lead(II) bromide (PbBr_2_, 99.999%, Sigma-Aldrich (St. Louis, MO, USA)), caesium bromide (CsBr, 99.999%, Sigma-Aldrich), oleic acid (OA, 99%, Sigma-Aldrich), oleylamine (OAm, 95%, SRL (Mumbai, India)), triotylphosphine (TOP, 97%, Sigma-Aldrich), 1-octadecene (ODE, 90%, Sigma-Aldrich), dimethylformamide (DMF, 99%), poly(methyl methacrylate) (PMMA), and poly(N-vinylcarbazole) (PVK) were purchased from commercial suppliers. All materials were used without further purification. The PL spectra were obtained using the Ocean Optics HR4000CG-UV-NIR spectrometer (Ocean Optics, Inc., Orlando, FL, USA).

### 2.2. Synthesis of CsPbBr_3_ Quantum Dots

The CsPbBr_3_ quantum dots were synthesised by the hot-injection method, which allows rapid nucleation and controlled growth of nanocrystals. The overall synthesis procedure is schematically shown in Figure 1a–d. A total of 10 mL of 1-octadecene (ODE) was added to a 50 mL round-bottom flask, and then prepared PbBr_2_ was added. Then, 1 mL oleic acid (OA), 1 mL oleylamine (OAm) and 0.5 mL trioctylphosphine (TOP) were added to the reaction mixture.

The mixture was heated to 80–100 °C under constant magnetic stirring until PbBr_2_ was completely dissolved and a clear homogeneous solution was obtained. In this step, OA and OAm interacted with Pb^2+^ ions to form lead–ligand complexes, which enhanced the solubility of PbBr_2_ and stabilised the precursor solution. This step is illustrated in Figure 1a.

### 2.3. Preparation of CsBr Precursor Solution

Separately, 0.2 mmol of CsBr was dissolved in 1 mL of dimethylformamide (DMF) under continuous stirring to obtain the Cs precursor solution. The DMF was used because of its high polarity to facilitate the dissolution of CsBr. Prior to injection, the CsBr solution was heated up to approximately 100 °C in order to enhance its reactivity and minimise the temperature changes during the injection process. The preparation of the Cs precursor solution is shown in Figure 1b.

### 2.4. Hot Injection and Nucleation

After complete dissolution of the PbBr_2_ precursor, the reaction mixture was heated up to 100–110 °C. The preheated CsBr solution was then rapidly injected into the PbBr_2_ precursor solution with a syringe. The fast injection resulted in a supersaturated system that led to the instantaneous nucleation of CsPbBr_3_ nanocrystals. The nucleation process took place in 3–5 s, and CsPbBr_3_ quantum dots were formed in the reaction medium. This is shown in Figure 1c.

### 2.5. Crystal Growth and Reaction Quenching

After nucleation, the nanocrystal experienced a brief growth phase during which the crystal size increased and the surface ligands stabilised the particle surface. OA, OAm and TOP prevented uncontrolled aggregation and resulted in a narrow particle size distribution. The reaction was quenched quickly by immersing the reaction flask in an ice-water bath to stop further crystal growth and maintain the desired nanocrystal size. The abrupt temperature decrease halted the growth process and stabilised the formed CsPbBr_3_ quantum dots. This is illustrated in Figure 1d.

### 2.6. Role of Chemicals During Synthesis

The formation of CsPbBr_3_ quantum dots is influenced by precursor, solvent and surface ligand interactions. The precursor materials PbBr_2_ and CsBr supply the Pb^+^, Cs^+^, and Br^−^ ions that comprise the perovskite crystal structure. These ions interact to form the CsPbBr3 lattice. 1-octadecene (ODE) is a high-boiling solvent, provides a stable reaction medium at high temperatures and allows controlled nucleation and growth of nanocrystals. CsBr is a good solvent for dimethylformamide (DMF) and allows a fast injection of the Cs precursor into the reaction mixture. The surface ligands used are oleic acid (OA), oleylamine (OAm) and trioctylphosphine (TOP). These ligands interact with the surface of the growing particles, minimising surface defects and avoiding particle agglomeration. Thus, controlling the size, stability and optical properties of the obtained quantum dots is important.

## 3. Results

The PL response of the synthesised CsPbBr_3_ quantum dots manifests a peculiar bimodal emission behaviour that can be directly attributed to the interplay of nucleation and growth under kinetically constrained conditions (Figure 2). The two well-resolved emission bands, with a dominant peak at ~510–520 nm and a secondary high-energy feature at ~430–440 nm, are indicative of the simultaneous presence of nanocrystals in different quantum confinement regimes.

The green emission (~510–520 nm) is attributed to band-edge excitonic recombination in relatively larger nanocrystals. This dual-emission signature is thus not a result of uncontrolled size dispersion but instead is a consequence of kinetic growth arrest caused by ultrafast reaction termination (3–5 s) and rapid quenching. These conditions decouple nucleation and growth in a way that enables partial retention of multiple size domains in a single synthesis window. The ratio of the relative intensities of the two emission bands further suggests that the synthesis is biased toward the formation of larger nanocrystals while maintaining a controlled fraction of strongly confined particles. The observed PL behaviour demonstrates reaction-time engineering as a direct approach to modulating the size-dependent emission wavelength, providing a tunable platform for the optical properties at the nanoscale.

### 3.1. Kinetic Model and Size Estimation

To quantitatively rationalise the observed reaction-time-dependent emission shift, we employ the classical LaMer model, which describes the hot-injection synthesis as a sequence of a rapid burst nucleation followed by a slower diffusion-limited growth stage [20]. In the diffusion-limited regime, the nanocrystal radius increases in proportion to the square root of the growth time. The size-dependent emission energy is then described by the Brus equation:

$$E\left(r\right)=E_{g}+\frac{h^{2}}{8r^{2}}\left(\frac{1}{m_{e}^{*}}+\frac{1}{m_{h}^{*}}\right)-\frac{1.786e^{2}}{4\pi \varepsilon \varepsilon_{0}r}$$

where $E_{g}\;$ is the bulk bandgap, $m_{e}^{*}\;and\;m_{h}^{*}$ are the effective masses of the electron and hole, ε is the dielectric constant, and r is the nanocrystal radius. The emission wavelength λ is related to E by λ = 1240/E (with E in eV and λ in nm).

For CsPbBr_3_, we use the following parameters: $E_{g}$ = 2.36 eV, $m_{e}^{*}=\;m_{h}^{*}=0.15\;m_{0}$, and ε = 6.5. Substituting the experimentally observed emission peaks (493, 505, and 515 nm) into the Brus equation, we obtain the corresponding nanocrystal radii: approximately 4.5 nm, 5.5 nm, and 6.9 nm, respectively. The estimated size increase of about 2.4 nm across the growth window fully accounts for the 22 nm spectral shift, confirming that the emission tunability originates from quantum confinement controlled by reaction time rather than from compositional changes.

### 3.2. Reaction-Time-Dependent Evolution of Photoluminescence Spectra of CsPbBr_3_ Quantum Dots Synthesised via Hot-Injection Method

The PL spectra of the synthesised CsPbBr_3_ quantum dots demonstrate a well-defined and progressive dependence on reaction time, as illustrated in Figure 3. Three post-injection growth intervals were investigated: 3–5 s (red), 5–10 s (green), and 10–20 s (blue). Among these, the first 10 s following hot injection represents the most decisive and optically sensitive phase of the entire synthesis process. During the 3–5 s interval, the reaction remains predominantly in its nucleation stage, yielding the smallest nanocrystals with the most pronounced quantum confinement. The strong spatial localisation of charge carriers at this stage elevates the effective bandgap significantly above the bulk value, producing a sharp emission centred near 493 nm accompanied by the highest PL intensity observed across all growth windows. Upon extending the reaction to the 5–10 s interval, nanocrystals enter a rapid growth phase driven by monomer addition onto existing nuclei. The consequent increase in particle size progressively relaxes the confinement potential, reducing the effective bandgap and red-shifting the emission toward 505 nm. Despite the reduction in peak intensity relative to the earliest stage, the quantum dots formed within this window retain strong radiative recombination efficiency, suggesting that surface passivation remains largely intact and non-radiative trap-assisted losses are still effectively suppressed.

Beyond the 10 s threshold, the reaction transitions into a slower, diffusion-limited growth regime as available precursor concentration diminishes and the system approaches thermodynamic equilibrium. In the 10–20 s interval, the emission peak shifts further to approximately 515 nm, while the PL intensity undergoes a more pronounced reduction compared to the earlier growth stages. This behaviour reflects the simultaneous contribution of three factors: an increase in average nanocrystal size, a broadening of the particle size distribution leading to inhomogeneous spectral broadening, and the progressive formation of surface defect states that introduce competing non-radiative recombination pathways. The systematic evolution of emission wavelength and intensity across all three growth windows confirms that the structural and optical characteristics of CsPbBr_3_ quantum dots are fundamentally established within the first 10 s of reaction. This finding underscores the critical importance of precise reaction-time control during hot-injection synthesis and establishes a reliable and highly reproducible strategy for engineering size-dependent emission across the blue–green spectral region for targeted optoelectronic and sensing applications.

### 3.3. Temperature-Dependent Photoluminescence Behaviour of PVK-CsPbBr_3_ Composites

The temperature-dependent response of PVK-embedded CsPbBr_3_ quantum dots is characterised by a stability in emission energy and a striking decrease in radiative output. As shown in Figure 4, the emission peak position is nearly constant in the range of 23–80 °C, approximately at 515–520 nm, which is attributed to the band-edge electronic structure or compositional fluctuations.

The PL intensity shows a systematic and pronounced decrease with increasing temperature, signalling the gradual activation of thermally assisted non-radiative recombination pathways. Such behaviour is dictated by an increased exciton–photon interaction which increases the probability of carrier relaxation into non-radiative channels. Further, interfacial electronic coupling between PVK and the quantum dots may provide an additional pathway for dissipation of carriers, which can contribute to quenching of the emission. Therefore, the thermal response of the PVK-CsPbBr_3_ system is governed by a change in recombination dynamics rather than variations in the intrinsic emission energy landscape.

### 3.4. Temperature-Dependent Photoluminescence Behaviour of PMMA-CsPbBr_3_ Composites

The thermal response of PMMA-embedded CsPbBr_3_ quantum dots is characterised by a progressive attenuation of emission intensity while preserving spectral position. As illustrated in Figure 5, the PL peak remains centred at approximately 505–510 nm across the temperatures.

As opposed to invariant emission energy behaviour, a systematic behaviour in PL intensity is observed with increasing temperature, reflecting the activation of thermally induced non-radiative pathways. The reduction in emission intensity can be attributed to enhanced exciton–photon interactions; the PMMA matrix, being electrically insulating, primarily acts as a physical barrier that suppresses environmental degradation while minimising interfacial charge transfer.

### 3.5. Comparative Analysis of PMMA and PVK-Embedded CsPbBr_3_ Quantum Dots

Comparison of the temperature-dependent PL behaviour in Figure 4 (PVK) and Figure 5 (PMMA) shows clear differences in the role of polymer matrices on the carrier recombination dynamics. Although both systems show stable emission positions with negligible spectral shift over the examined temperature range, their intensity evaluation under thermal variation differs significantly. As shown in Figure 4, the quenching of the PL intensity with increasing temperature is more severe for the PVK-embedded CsPbBr_3_ quantum dots, whereas the PMMA-embedded system (Figure 5) still exhibits a relatively higher emission intensity at the same thermal conditions. 

This contrast arises from the differences in the electronic nature of the polymer hosts, which are inherent in their electronics. As an electrically insulating matrix, PMMA mainly offers physical confinement and environment protection, thus maintaining the intrinsic radiative recombination processes of the quantum dots. In contrast, PVK, a semiconducting polymer, causes interfacial electronic interactions that can promote further non-radiative recombination pathways via charge transfer or carrier trapping. Figure 4 shows that the thermal response is affected by intrinsic exciton–phonon coupling and interfacial carrier dynamics, while Figure 5 shows that the response is mainly dominated by intrinsic thermal relaxation processes. This comparison highlights the major role of polymer–quantum dot interactions in tuning the temperature-dependent optical response of hybrid nanocomposites.

### 3.6. Emerging Sensor Applications

The quantum dots of CsPbBr_3_ prepared in this work provide a single-composition platform whose optical properties can be deliberately engineered through the precise control of reaction time. This allows for the design of sensor materials in which the sensing properties of the material are not inherent in its chemical identity but are actively programmed in during the synthesis process itself. The physical origin of this programmability is the quantum confinement effect. In contrast to bulk semiconductors, where the energy levels are continuous, when the physical dimensions of a semiconductor nanocrystal are reduced below the exciton Bohr radius of the material, the energy levels of the system become discrete and strongly size-dependent. With the reduction in the size of the nanocrystal, the spatial confinement of the charge carriers increases, and the energy levels of the electron and the hole are pushed further apart, such that the effective bandgap of the material is larger than its bulk value. Thus, controlling the nanocrystal size directly controls the bandgap, and controlling the bandgap directly controls the wavelength of the emission. In the hot-injection synthesis used in this work, the size of the nanocrystals is primarily controlled by the length of the post-injection growth step. As shown in Figure 2, increasing the growth interval from 3–5 s to 5–10 s and to 10–20 s results in a gradual increase in the size of the nanocrystals and a continuous shift in the emission peaks from 493 nm to about 515 nm. The 22 nm tunable spectral window achieved without any change in precursor chemistry, reaction temperature or material composition is a direct consequence of reaction-time-dependent bandgap engineering and the core capability of this material system for sensor applications.

Most importantly, the first 10 s after hot injection is found to be the most optically sensitive and controllable stage of the whole synthesis. In this narrow time window, the relationship between reaction duration and emission wavelength is the steepest, so that even small differences in quenching time lead to measurable and reproducible changes in the optical output of the resulting quantum dots. This extreme sensitivity of optical properties to early-stage growth kinetics presents both a challenge and an opportunity. It is a challenge in that it requires precise experimental control of injection conditions and quench timing. This is an opportunity because the emission wavelength of the final product can be finely tuned with high resolution by simply adjusting the point at which the reaction is stopped, with no further processing steps. This directly translates into the ability to design sensors that can be engineered to produce QDs with a given emission wavelength that is pre-matched to the spectral requirements of a given sensing target. This opens up the possibility of developing wavelength-selective optical sensors that can discriminate between different analytes or environmental conditions simply on the basis of their spectral signature.

The other significant sensing ability demonstrated here comes from the temperature dependence of the PL behaviour of the polymer-embedded quantum dot systems, as shown in Figure 4 and Figure 5. Both PVK- and PMMA-embedded systems show a systematic and monotonic decrease in PL intensity with increasing temperature within the temperature range examined (23–80 °C), while the emission peak position remains spectrally invariant. This behaviour is physically important since it implies that the temperature information is encoded only in the intensity of the emitted light and not in its wavelength. This is very advantageous for a sensing system since intensity-based measurements are simpler to implement, need less-complex detection instrumentation and can be performed completely without physical contact with the object being measured. The result is a temperature-responsive luminescence platform, where temperature can be simply read out by monitoring the brightness of the PL signal from the quantum dot composite film.

The choice of polymer matrix introduces another powerful degree of freedom to this sensing platform. The PMMA-embedded system functions primarily as a physical encapsulant that protects the quantum dot surface from environmental degradation with little perturbation of the intrinsic recombination dynamics of the nanocrystals due to the electrically insulating nature of the polymer host. This property gives a thermal response dominated by intrinsic exciton–phonon coupling in the quantum dots themselves, resulting in a moderate, stable and highly reproducible intensity variation with temperature. This property makes the PMMA composite very suitable for applications demanding reproducibility of measurement over a long period of time and reliable baseline performance, e.g., for the continuous measurement of environmental temperature or process control in industry. On the contrary, the PVK-embedded system brings a totally different physical mechanism to the thermal response. PVK is a semiconducting polymer with intrinsic charge transport properties, and the addition of CsPbBr_3_ quantum dots forms interfacial electronic coupling between the polymer and the surface of the nanocrystal that offers additional pathways to thermally excited carriers that escape from the quantum dot without photon emission. The interfacial non-radiative channels are strongly activated with temperature, resulting in a much sharper and more pronounced decrease in the PL intensity per degree of the increase in temperature than in the PMMA system. Such increased sensitivity makes the PVK composite an ideal candidate for applications that require the detection of small temperature variations with high resolution, such as biomedical temperature sensing, microelectronic thermal management, or precise laboratory instrumentation.

The true revolution of this platform for the future of sensing technology is the coexistence of multiple independently tunable sensing parameters within a single material system. The emission wavelength can be tuned by controlling reaction time during the synthesis. Thermal sensitivity can be tuned by choosing an appropriate polymer matrix. The mechanical form factor can be designed by casting the composite into flexible films. The entire system is operated solely via optical transduction, with no electrical contacts, no moving parts and no direct physical connection to the object being measured. This combination of the properties of spectral programmability, thermally responsive luminescence, polymer tunable sensitivity and mechanical flexibility is simply not available in any conventional sensor technology. Thermocouples and resistance temperature detectors are widely used but are rigid by nature, require direct physical contact, and have no spectral tunability whatsoever. Conventional optical sensors that rely on bulk semiconductors or organic dyes suffer from several drawbacks, such as fixed emission wavelengths, low thermal stability, and incompatibility with flexible substrates. The CsPbBr_3_ QD–polymer composites developed in this work simultaneously resolve all of these limitations, establishing a new material paradigm for the design of optically programmable, thermally stable, spectrally tunable, and mechanically flexible sensing platforms for applications ranging from non-contact thermometry and wavelength-selective chemical detection to wearable health monitoring and next-generation photonic sensing architectures.

## 4. Discussion

This study presents the findings that offer several important insights into the optical behaviour of CsPbBr_3_ quantum dots and their potential as functional sensing materials. The results are discussed here in the context of established theoretical frameworks, relevant prior investigations and the broader implications for the field of quantum dot-based optical sensing.

### 4.1. Reaction-Time-Dependent Bandgap Engineering and Emission in Size-Controlled

By varying the reaction time, we find a continuous and reproducible shift in the emission wavelength from 493 nm to 515 nm, which is in agreement with the well-established quantum confinement theory. The theory predicts an inverse relationship between the nanocrystal size and the effective bandgap in semiconductor systems where the dimensions are below the exciton Bohr radius. The results of this work, however, are more than just a confirmation of this theoretical expectation. The identification of the first 10 s post-injection as the most optically sensitive phase of nanocrystal formation provides a previously underappreciated level of temporal resolution to understanding the nucleation and growth kinetics of CsPbBr_3_ nanocrystals. Previous studies have demonstrated that the synthesis of perovskite quantum dots by hot injection proceeds with an extremely rapid nucleation burst followed by a relatively slow growth phase, but the optical consequences of early growth termination at the sub-10 s time scale have not been systematically investigated. The present results unambiguously demonstrate that this early growth window is not a mere transient intermediate stage but rather the defining moment at which the spectral identity of the final quantum dot product is irreversibly established. This finding has direct and immediate implications for the synthesis reproducibility and scalability of quantum dots, highlighting the critical importance of precise injection timing and quenching control in achieving consistent optical output across synthesis batches.

Moreover, achieving a 22 nm tunable spectral window in a single, unmodified material composition is a practically significant step forward compared to halide composition engineering-based methods to achieve spectral tunability. While halide substitution in CsPbX_3_ systems provides a wider tunable range in principle, it introduces substantial additional complexity in terms of phase stability, ion migration and reproducibility of optical properties. The kinetic tunability demonstrated in this work circumvents these complications altogether, providing a simpler, more stable and reproducible pathway to targeted emission wavelength control that is directly translatable to scalable manufacturing processes.

### 4.2. Temperature-Dependent Optical Response and Thermal Quenching Mechanism

The systematic decrease in PL intensity with temperature as observed in bare, PMMA-embedded and PVK-embedded quantum dot systems is in full agreement with the established understanding of thermally activated non-radiative recombination in semiconductor nanocrystals. At elevated temperatures, the phonon population increases, and so does the likelihood of an exciton–phonon scattering event. This provides sufficient energy for the thermally excited carriers to overcome the potential barrier between radiative recombination states and non-radiative trap states at or near the surface of the nanocrystal. The observed monotonic decrease in the PL intensity with temperature is due to the consequent redistribution of the carrier population away from radiative channels. 

It is especially important that the position of the emission peak is spectrally invariant over the entire temperature range studied for all the systems investigated. This behaviour is opposite to that seen in many conventional semiconductor quantum dot systems, where thermal expansion of the crystal lattice and renormalisation of the electronic band structure under elevated temperature conditions leads to a measurable red-shift in emission wavelength with increasing temperature. The absence of such a spectral shift in the CsPbBr_3_ systems studied here suggests that the perovskite crystal lattice and electronic band structure are structurally and compositionally robust over the temperature range studied and that the thermal effects are manifested only through the carrier dynamics, rather than any change in the fundamental material properties. Such spectral stability is a critically important practical advantage for sensing applications, because it guarantees that temperature measurements based on emission intensity are not confounded by concurrent wavelength shifts that would otherwise introduce systematic errors into the optical readout.

### 4.3. Role of Polymer Matrices in Modulating Thermal Optical Response

The observed difference in thermal behaviours of the PMMA- and PVK-embedded systems provides us important insights into the physical mechanisms by which polymer host environments influence the carrier recombination dynamics of embedded quantum dots. The improved emission stability of the PMMA composite is in agreement with the known effect of an insulating polymer matrix to prevent formation of surface trap states and block penetration of moisture and oxygen, which would otherwise promote non-radiative recombination. PMMA also efficiently freezes the intrinsic radiative recombination character of the nanocrystals by physically isolating the quantum dot surface from the environment. The PL response of the nanocrystals can then evolve with temperature according to intrinsic exciton–phonon coupling alone without being affected by extrinsic environmental factors.

The PVK composite shows an increased thermal sensitivity, and this brings a more complex and physically richer set of considerations. The electronic coupling at the interface of the semiconducting PVK host with the surface of the CsPbBr_3_ quantum dot establishes a hybrid electronic environment in which thermally excited carriers can relax non-radiatively outside the boundaries of individual nanocrystals. This behaviour is similar in concept to the charge transfer quenching mechanisms reported in organic–inorganic hybrid systems, where the energy alignment between the donor and acceptor states at the heterointerface controls the efficiency of the non-radiative transfer of carriers. The temperature dependence of this interfacial charge transfer process enhances the overall thermal quenching response of the PVK composite over the intrinsic exciton–phonon coupling, resulting in the enhanced sensitivity that distinguishes this system from its PMMA counterpart. This mechanistic insight provides a rational basis for the deliberate design of quantum dot–polymer composites with tailored thermal sensitivity profiles, where the extent of interfacial electronic coupling can be tuned through selection of the polymer host to achieve a desired balance of emission stability and temperature sensitivity.

Another mechanism prominent in QD–polymer interactions is the formation of polarons. Perovskite nanocrystals are known to exhibit unusually strong exciton–phonon coupling compared with conventional II–VI and III–VI quantum dots, to the extent that single-exciton lattice distortions (exciton-polarons) have been directly resolved in CsPbBr_3_ QDs [21]. Huang-Rhys analyses of glass-embedded CsPbI_3_ nanocrystals further show measurable exciton–phonon coupling strength and long polaron coherence time [22], and self-trapped excition-polaron states have been reported to produce asymmetric emission lineshapes in two-dimensional CsPbBr_3_ perovskites [23].

In conventional semiconductor quantum dots, coupling of a confined exciton to phonons of the surrounding host medium is known to produce a slight shift in the emission resonance and to contribute to the dephasing of the excitonic state [24]. In the present PMMA and PVK composites, the emission peak position was found to be spectrally invariant across the 23–80 °C range (Figure 4 and Figure 5), suggesting that any polaronic energy renormalisation induced by the host matrix is either negligible or masked within our spectral resolution over this temperature window. We note, however, that our measurements were intensity-resolved rather than coherence- or linewidth-resolved, and cannot rule out a polaronic contribution to dephasing or homogeneous spectral broadening that would not manifest as a shift in the ensemble PL peak position. Distinguishing exciton–phonon coupling to the QD’s intrinsic lattice from coupling mediated by the polymer host for example via temperature-dependent linewidth analysis or low-temperature photon-echo spectroscopy represents an important direction for future work, particularly given the differing electronic character of PMMA and PVK.

### 4.4. Implications for Optical Sensing and Beyond

Taken together, the results of this study have several important implications for the practical development of optical sensing platforms (Figure 6). The demonstration of temperature-sensitive luminescent response using CsPbBr_3_ quantum dot–polymer composites is in line with a growing body of research investigating luminescent materials as alternatives to conventional contact-based temperature measurement technologies. But the present work surpasses the investigations existing in two important respects. First, the reaction-time-controlled spectral tunability combined with polymer-mediated sensitivity engineering on a single material platform provides a level of functional versatility that has not been demonstrated before in CsPbBr_3_-based sensing systems. Second, the systematic comparative analysis of PMMA- and PVK-embedded systems provides a physically grounded design framework for selection and optimisation of polymer matrix composition based on the specific sensitivity and stability requirements of a given sensing application.

In addition to temperature sensing, the spectral programmability demonstrated here provides several promising directions for future work. The possibility of synthesising quantum dots with emission wavelengths pre-matched to the absorption features of particular chemical analytes indicates possible applications in wavelength-selective optical chemical sensors and biosensing platforms. Moreover, the compatibility of the quantum dot–polymer composite films with flexible substrate processing offers a promising direction for integrating these materials into wearable sensing devices, which should be specifically explored in future work. It would also be of value to extend the temperature-dependent PL analysis to lower temperatures and wider temperature ranges to establish the full operational envelope of these sensing platforms and to provide a more detailed insight into the low-temperature carrier dynamics of polymer-embedded CsPbBr_3_ quantum dots.

While the present study demonstrates a monotonic temperature-dependent decrease in PL intensity with no spectral shift, a fully quantitative thermometric platform would require additional calibration. Key metrics include the relative thermal sensitivity, the temperature resolution, and the activation energy extracted from an Arrhenius plot. Such analyses have been reported for other perovskite QD–polymer composites [25], where thermal sensitivity values of a few % per °C and sub-degree resolution were achieved. In our system, the stronger quenching observed for PVK compared to PMMA suggests that PVK-based composites may offer higher sensitivity, while PMMA provides better stability and reproducibility. Establishing full calibration curves for these materials is a natural next step and will be pursued in future work.

The results presented here provide a strong foundation for the development of CsPbBr_3_ quantum dot–polymer composite sensing platforms, but several key questions remain and are worthy of systematic investigation in future work. The long-term photostability of the quantum dot composites under continuous optical excitation has not been addressed in the present study and represents a critical parameter for practical sensing deployment. A quantitative study of the effect of the film thickness and the loading concentration of the quantum dots on the thermal sensitivity and the emission stability of the composite systems is needed to determine the optimal fabrication parameters. The use of ratiometric sensing schemes, where the sensing signal is the ratio of the emission intensities at two different wavelengths rather than the absolute intensity, would significantly enhance the measurement accuracy and environmental robustness of these platforms by removing the influence of excitation intensity fluctuations on the optical readout. Finally, the exploration of alternative polymer matrices with intermediate electronic properties between fully insulating PMMA and semiconducting PVK might enable composite systems with finely tunable sensitivity profiles, further expanding the design space available for engineering quantum dot-based optical sensors with application-specific performance characteristics.

## 5. Conclusions

In this paper, we propose a coherent and experimentally validated paradigm for rational design of optically programmable CsPbBr_3_ quantum dot sensing platforms that shows that the spectral and thermal properties of perovskite nanocrystals are not fixed material properties but rather precisely engineer able quantities accessible by deliberate control of synthesis kinetics and polymer host selection. A key conclusion of this work is that reaction time during hot-injection synthesis is a vital synthesis parameter that regulates the optical properties of CsPbBr_3_ quantum dots by controlling nanocrystal size and the resulting quantum confinement effect.

The nanocrystal size, quantum confinement strength, effective bandgap and PL emission wavelength are set simultaneously and irreversibly within the first 10 s after the injection of the precursors. Modulating the reaction time without changing the precursor chemistry or reaction temperature, a continuous red-shift in emission from 493 nm to 515 nm was obtained. This is strong experimental evidence that growth kinetics, rather than thermodynamic equilibrium, dominates spectral tunability in this material system, which is a conceptual departure from the traditional composition-dependent approaches to bandgap engineering in quantum dot systems. Temperature-dependent PL measurements in the temperature range from 23 to 80 °C showed a thermally activated gradual decrease in the emission intensity without any observable change in the emission peak wavelength. We attribute this decoupling of the radiometric output from the spectral position to the selective activation of non-radiative recombination pathways due to the enhanced exciton–phonon coupling, independent of any alteration of the fundamental electronic band structure or the material stoichiometry.

This behaviour is an intrinsically self-referencing transduction mechanism that has potential for thermometry applications, where temperature is encoded in the luminescence signal without spectral ambiguity. We demonstrate that the host environment actively controls the thermal optical response of the embedded nanocrystals, as evidenced by a systematic investigation of PMMA and PVK polymer composite matrices. The insulating nature of PMMA physically decouples quantum dot surfaces from interfacial charge exchange, preserving intrinsic radiative recombination dynamics, and providing a thermally stable, reproducible optical response, which is well suited to long-term sensing applications. In contrast, the semiconducting nature of PVK introduces the interfacial electronic coupling which significantly enhances the thermal quenching and, thus, results in greatly enhanced thermal sensitivity and fast response characteristics, which are beneficial for high-resolution precision thermometry. Taken together, these results demonstrate the convergence of reaction-time-controlled bandgap engineering, polymer-mediated thermal sensitivity modulation, and intensity-based optical transduction into a single material platform with compositional unity. The main advantage of this approach is the independent control of the spectral output, thermal sensitivity, and mechanical form factor by well-defined and scalable engineering variables, providing a strong foundation for the development of next-generation spectrally programmable photonic sensors and mechanically flexible wearable devices. Therefore, CsPbBr_3_ quantum dot–polymer composites constitute a practically feasible and versatile platform for advanced optical sensing applications.

## Figures and Tables

**Figure 1:**
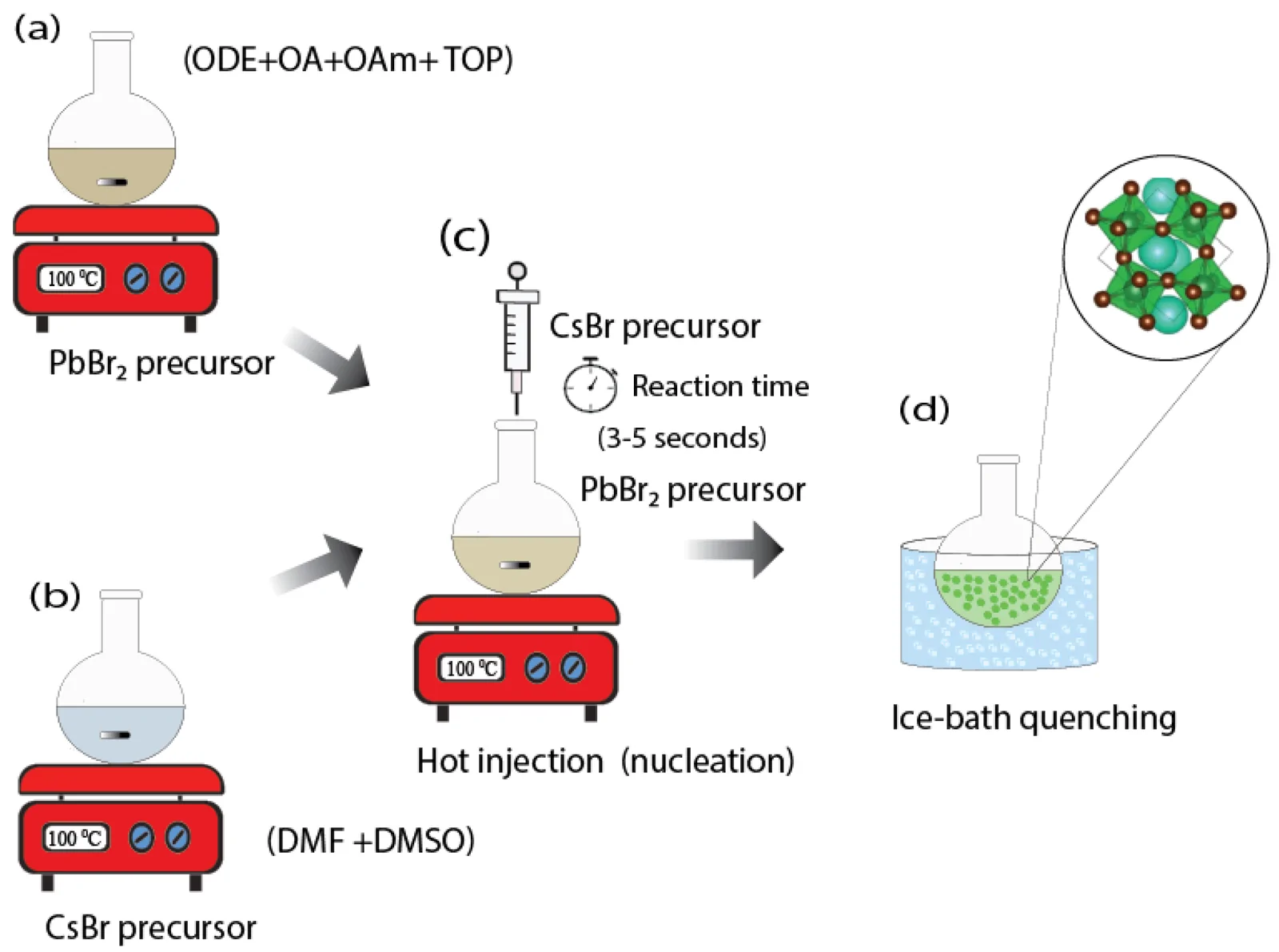
Schematic of the nucleation and growth process: (**a**) PbBr_2_ precursor preparation, (**b**) CsBr precursor injection, (**c**) short reaction time (3–5 s), and (**d**) ice-bath quenching.

**Figure 2:**
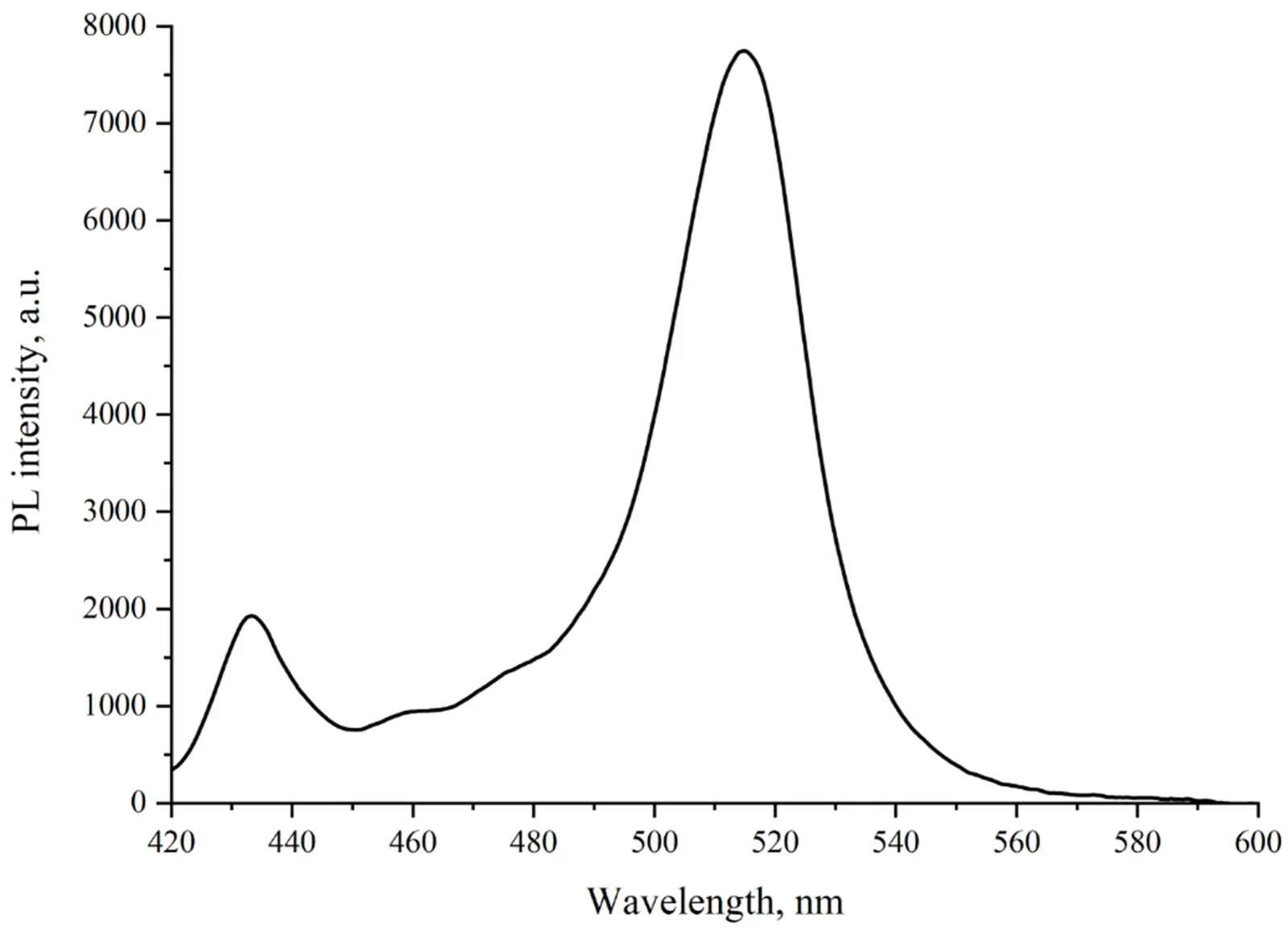
Room-temperature photoluminescence spectrum of size-controlled CsPbBr_3_ quantum dots synthesised via hot-injection method.

**Figure 3:**
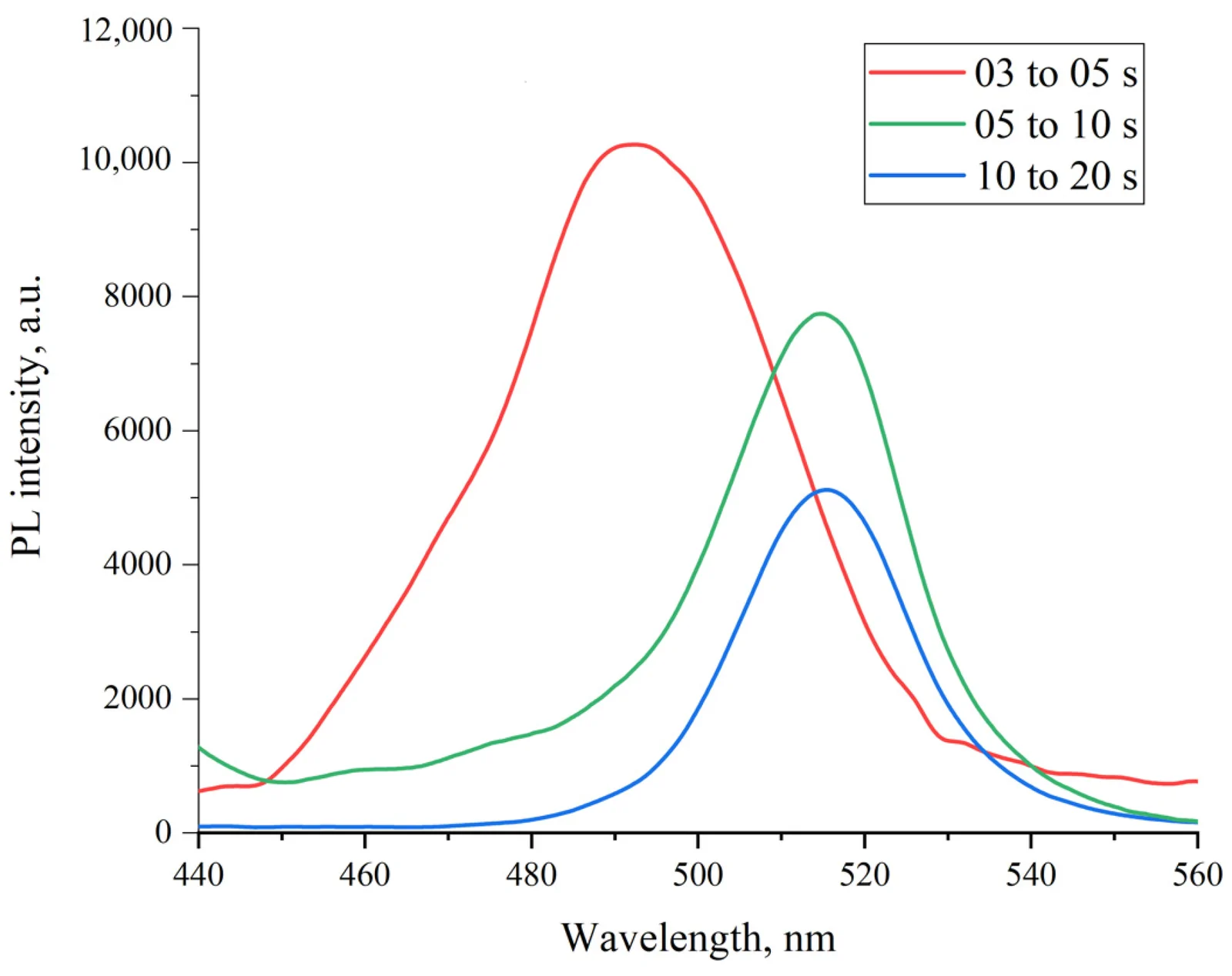
Photoluminescence emission spectra of CsPbBr_3_ quantum dots synthesised with different reaction times (3–5 s, 5–10 s, and 10–20 s), demonstrating size-dependent tuning of the emission wavelength via the quantum confinement effect.

**Figure 4:**
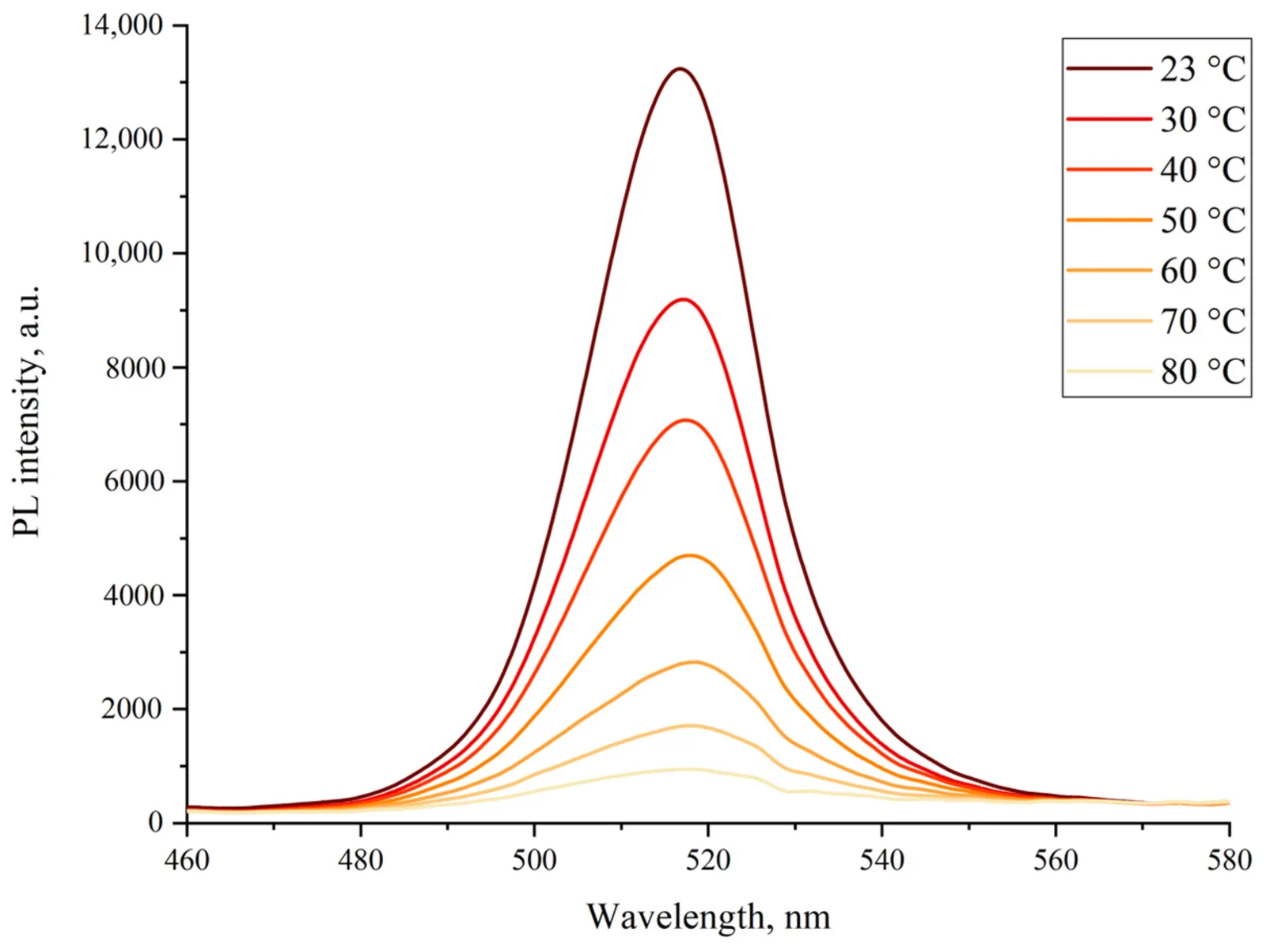
Evolution of photoluminescence spectra of PVK-encapsulated CsPbBr_3_ quantum dots under varying temperature (23–80 °C).

**Figure 5:**
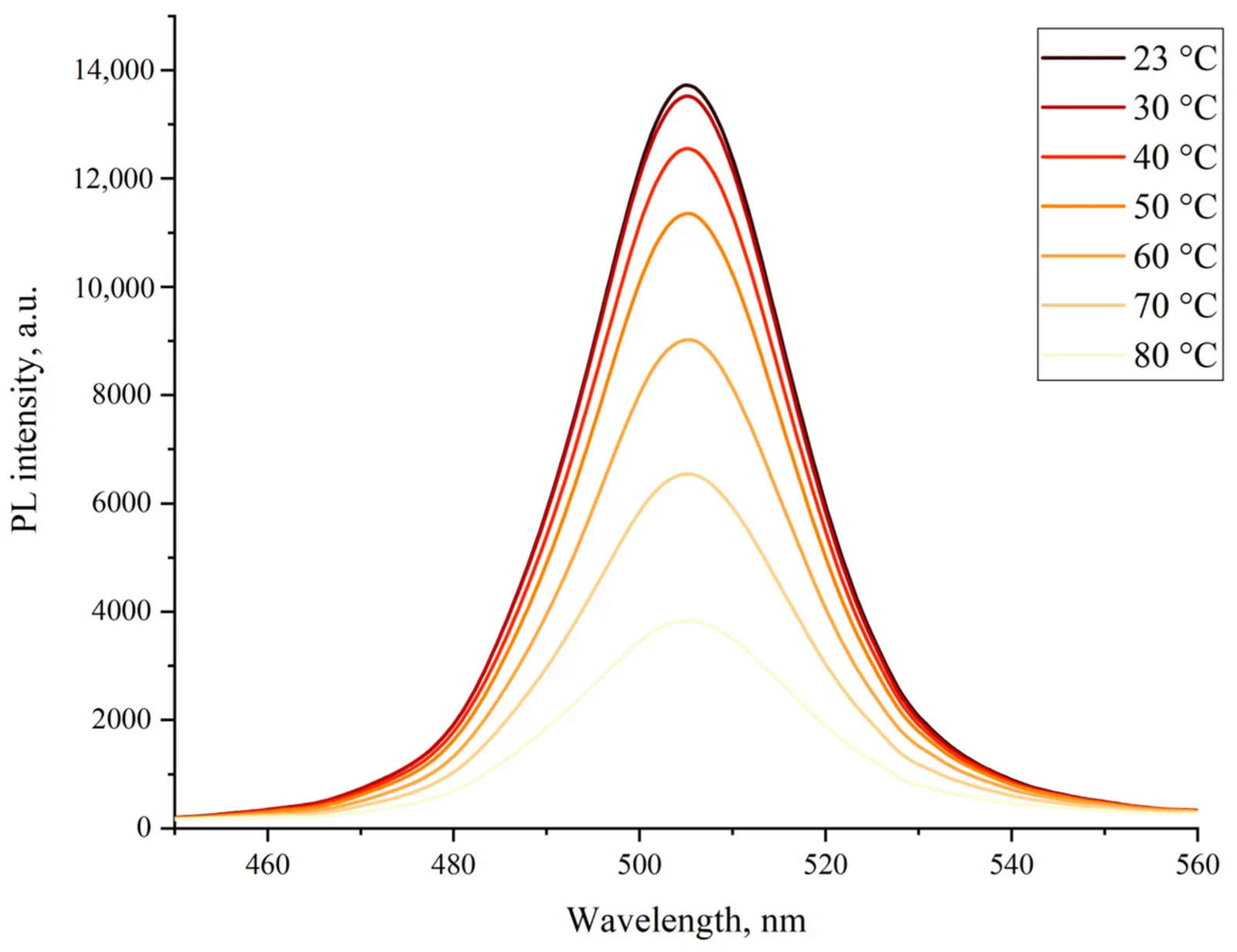
Evolution of photoluminescence spectra of PMMA-embedded CsPbBr_3_ quantum dots under varying temperature (23–80 °C).

**Figure 6:**
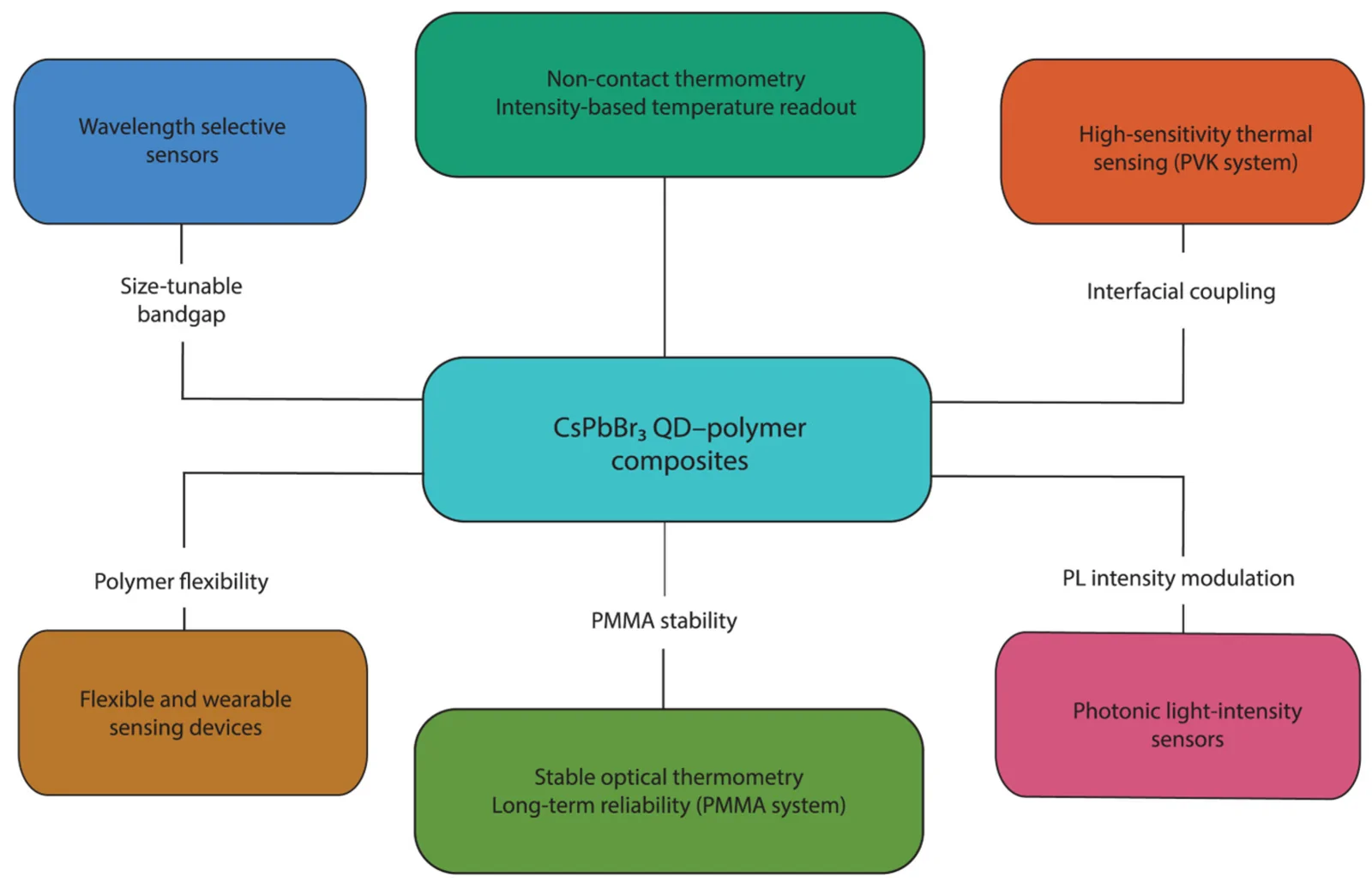
Emerging sensor applications of CsPbBr_3_ QD–polymer composites.

## Data Availability

The data that support the findings of this study are available from the corresponding author upon reasonable request.

## Abbreviations

The following abbreviations are used in this manuscript:

QD	Quantum Dot
PQDs	Perovskite Quantum Dots
CsPbBr_3_	Caesium Lead Bromide
PL	Photoluminescence
PLQY	Photoluminescence Quantum Yield
PMMA	Poly(methyl methacrylate)
PVK	Poly(N-vinylcarbazole)
OA	Oleic Acid
OAm	Oleylamine
TOP	Trioctylphosphine
ODE	1-Octadecene
DMF	Dimethylformamide
LED	Light-Emitting Diode
UV	Ultraviolet
XRD	X-ray Diffraction
TEM	Transmission Electron Microscopy
UV-Vis	Ultraviolet–Visible Spectroscopy
FWHM	Full Width at Half Maximum
CdSe	Cadmium Selenide
InP	Indium Phosphide
